# How Does Time Spent Working in Custody Influence Health and Fitness Characteristics of Law Enforcement Officers?

**DOI:** 10.3390/ijerph18179297

**Published:** 2021-09-03

**Authors:** Robert G. Lockie, Karly A. Rodas, J. Jay Dawes, Joseph M. Dulla, Robin M. Orr, Matthew R. Moreno

**Affiliations:** 1 Department of Kinesiology, California State University, Fullerton, Fullerton, CA 92835, USA; cesariokarly@csu.fullerton.edu (K.A.R.); moreno.matthewr@csu.fullerton.edu (M.R.M.); 2 School of Kinesiology, Applied Health and Recreation, Oklahoma State University, Stillwater, OK 74078, USA; jay.dawes@okstate.edu; 3 Tactical Research Unit, Bond University, Robina, QLD 4229, Australia; joseph.dulla@student.bond.edu.au (J.M.D.); rorr@bond.edu.au (R.M.O.)

**Keywords:** aerobic fitness, blood pressure, correctional, deputy sheriff, fat mass, muscular endurance, police, resting heart rate, tactical, YMCA step test

## Abstract

This study investigated the influence of time spent working in custody on the health and fitness of law enforcement officers (LEOs). Retrospective analysis was conducted on data from 48 male and 12 female LEOs, divided into groups based upon time spent working custody: LEO ≤ 24 (≤24 months; *n* = 15); LEO 2547 (25–47 months; *n* = 24); and LEO 48+ (≥48 months; *n* = 21). The following were measured: body mass index (BMI); fat mass percentage; waist-to-hip ratio (WHR); resting heart rate (RHR); blood pressure; grip strength; sit-and-reach; push-ups; sit-ups; and YMCA step test recovery heart rate (HR). A univariate ANCOVA (controlling for sex and age) with Bonferroni post hoc determined significant between-group differences. Select assessments were compared to normative data. The LEO 48+ group completed fewer sit-ups than the LEO 2547 group (*p* = 0.006); there were no other significant between-group differences. Forty-nine LEOs were overweight or obese according to BMI; 52 were fatter than average or above; 27 had a WHR that increased cardiovascular disease risk. Forty-three LEOs had very poor RHR; 52 had elevated blood pressure. Forty-eight LEOs had average-to-very poor step test recovery HR. Irrespective of time spent working in custody, personnel should be physically active to maintain health and fitness and, where possible, engage in formal strength training and conditioning.

## 1. Introduction

Policing and law enforcement can be very physically and mentally demanding. Law enforcement officers (LEOs) need to perform numerous job-specific tasks when on duty, which can range from sedentary, low-intensity actions (e.g., sitting in a vehicle, office work), to high-intensity activities (e.g., pursuing and apprehending offenders) [1,2]. Although the sedentary activities may predominate [2], the ability to complete the high-intensity actions could determine an officer’s ability to ensure safety of the public, themselves, and their colleagues. As a result, recruits will typically complete academy training to physically and mentally prepare for the occupational demands of policing [3,4]. Given the relationships between fitness and job-specific task performance [4,5,6,7,8,9], it would be ideal for recruits to be highly fit prior to being deployed for duty as an LEO. Indeed, several studies have indicated that recruits can experience improvements in fitness qualities such as muscular strength, power, endurance, and aerobic capacity following academy training [3,10,11].

A patrol position is where recently trained LEOs would be able to perform the occupational skills they developed during academy. However, for many LEOs from law enforcement agencies in the USA, the first position immediately following academy is working in custody facilities [12,13]. Custody facilities include jails, detention, or court lockup facilities [14]. The primary job tasks when working in custody are different to those required for patrol, and tend to be even more low intensity [15,16]. For example, some of the major job tasks when working in custody include office work, processing and supervising inmates, and cell searches [15,16]. Depending on the agency, LEOs could work in custody for several months to several years, depending on available patrol positions at the station to which they are assigned [12]. The length of time an LEO spends working in custody could have a major impact on an individual’s general and job-specific health and fitness.

There has been previous research investigating job-specific fitness changes in LEOs following working in custody [12,13]. Job-specific fitness was measured by Lockie et al. [12,13] via the Work Sample Test Battery (WSTB), which consists of five tasks: a 99-yard (90.53 m) obstacle course; a body drag with a 165-pound (74.84 kg) dummy; a climb over a six-foot (1.83 m) chain link fence; a climb over a six-foot solid wall; and a 500-yard (457.2 m) run. Lockie et al. [12] performed a cross-sectional analysis to assess WSTB performance changes in LEOs categorized by time spent working in custody compared to recruits. LEOs who had spent 47 months or less working in custody were slower compared to recruits in the 500R. LEOs who worked in custody for 48 months or more were slower in all WSTB tasks (except the body drag) compared to recruits and officers who had been working in custody for less time. In a longitudinal study, Lockie et al. [13] found that the time to complete the individual WSTB tasks increased by approximately 4–83% in male and female LEOs from the end of academy until their first patrol assignment (i.e., after their time spent working in custody). Time spent working in custody negatively impacted the job-specific fitness of LEOs [12,13]. From this research, it could be surmised that the general health and fitness of LEOs may be negatively impacted by custody work.

Noting these impacts on fitness, general health and fitness should also be a consideration for LEOs, as this could not only impact their ability to perform job tasks [4,5,7,8], but also their well-being and quality of life [17,18]. Previous research has suggested that the nature of patrol work can have negative impacts on an officer’s health and fitness [2,19]. Orr et al. [19] conducted a cross-sectional comparison between patrol officers and cadets during academy to analyze potential decrements in fitness. Orr et al. [19] found that male patrol officers performed 18% fewer push-ups and 15% fewer sit-ups in 60 s and were 16% slower in a 300 m and 2.4 km run (poorer muscular endurance, anaerobic endurance, and aerobic fitness, respectively) when compared to cadets. Female patrol officers lifted 21% less load in a one-repetition maximum bench press (upper-body maximal strength) and performed 36% fewer push-up repetitions in 60 s (upper-body muscular endurance) compared to cadets. Although there may be some age-related declines [20,21,22,23], Orr et al. [19] did link these fitness-related differences to the sedentary nature of the policing profession.

In addition to fitness, it is important to consider health-related indicators (e.g., blood pressure [BP], body composition, etc.) for LEOs working in custody. The nature of the law enforcement profession can increase the risk of cardiovascular disease in officers [24]. This relates to the cumulative effect of factors such as sedentary behaviors, shift work, long working hours, and stress [24,25]. Some practical assessments that could be used to indicate cardiovascular disease risk include resting heart rate (RHR) [26,27,28], BP [29], body mass index (BMI) [30], and body composition [30,31]. Indeed, some of these tests have been previously recommended to document the health characteristics of law enforcement populations [32]. As custody work involves potentially more sedentary activity than patrol duties [15,16], any negative changes in health and fitness for LEOs working in custody could be exacerbated by longer periods working in custody. However, there has been limited analysis of this within the literature.

Therefore, a cross-sectional analysis of LEOs was conducted to analyze the influence of time spent working in custody on measures of health and fitness. The study involved the analysis of de-identified archival data, which were provided to the researchers. Agency staff conducted health and fitness testing, which incorporated a range of assessments that have been recommended within the literature [32]. The LEOs were split into groups based upon their time spent working in custody to investigate whether this impacted their health and fitness [12]. In addition to the between-group comparisons, scatter plot data were produced to document individual LEO health and fitness, and data from the LEOs were compared to norms where appropriate [33,34,35]. Identifying whether LEOs are actually healthy and fit individuals relative to the general population when they are expected to work patrol is critical information to identify. This is because LEOs who may not be as healthy and fit as they should be could impact the safety of the officer, their colleagues, and the general population. It was hypothesized that LEOs who spent a longer time working in custody would have a poorer health and fitness profile compared to LEOs that worked in custody for a shorter time period. It was further hypothesized that even with the differences in time spent working in custody, the LEOs from this sample would have poorer health and fitness characteristics compared to normative data from the general population due to the sedentary nature of their duties.

## 2. Materials and Methods

### 2.1. Subjects

Archival data from one patrol school class from one law enforcement agency, comprising 60 LEOs (age: 32.03 ± 6.25 years; height: 1.72 ± 0.08 m; body mass: 86.46 ± 16.32 kg), which included 48 males (age: 30.83 ± 5.61 years; height: 1.75 ± 0.07 m; body mass: 91.33 ± 14.12 kg) and 12 females (age: 36.83 ± 6.65 years; height: 1.61 ± 0.03 m; body mass: 67.00 ± 7.72 kg), were analyzed. This was a convenience sample of de-identified data provided by the agency, and the researchers had no control of the final sample size used in this investigation. Inclusion criteria for the participants included complete datasets. Based on the retrospective nature of this analysis, the institutional review board approved the use of pre-existing data (HSR-17-18-370). Nonetheless, the study was still conducted according to the guidelines of the Declaration of Helsinki [36].

### 2.2. Procedures

The data were collected by staff working for one agency during patrol school. Patrol school was a three-week skills refresher program completed by LEOs who had been working in custody, as they did not complete any patrol duties during this time [12]. The health and fitness testing conducted by the agency was completed voluntarily by LEOs who opted into the assessment. Testing was conducted indoors on a basketball court at the agencies’ training facility in groups of 10–15. The staff involved with testing were all trained by a certified Tactical Strength and Conditioning Facilitator who verified the proficiency of the staff members. LEOs self-reported their age and time they had spent working in custody at the start of the session. RHR and BP were recorded first, followed by height, body mass, and percentage of fat mass (FM%). The LEOs then progressed through a testing circuit of waist and hip measurements, sit-and-reach, and grip strength. The LEOs completed push-ups and sit-ups as a group, before completing the YMCA step test last. The methods for each test are presented in the chronological order they were completed within the session.

### 2.3. Resting Heart Rate (RHR) and Blood Pressure (BP)

RHR and BP were recorded after the LEOs were seated quietly for approximately 5–10 min. Electronic BP monitors (Omron Healthcare, Kyoto, Japan) were utilized by this agency due to their ease of use, consistency, and need for time management during patrol school. The use of electronic BP monitors has been recommended by the Centers for Disease Control and Prevention [37], and by Rodas and Lockie [32] in law enforcement populations. LEOs were seated with their feet flat on the floor and their arm in a supported, relaxed position at heart level. Clothing was removed or repositioned such that the cuff was placed on bare skin without any compression above the cuff. The cuff position was above the crease of the elbow and encircled approximately 75–100% of the arm [38]. Staff then followed the directions presented on the automated device. RHR (measured in beats per minute; bpm), systolic BP, and diastolic BP were recorded. BP was measured in millimeters of mercury (mmHg).

### 2.4. Age, Height, Body Mass, Percentage of Fat Mass (FM%), and Body Mass Index (BMI)

Height was measured barefoot using a portable stadiometer (Seca 217, Hamburg, Germany). Body mass and FM% were recorded by electronic digital scales (Model HBF-510, Omron Healthcare, Kyoto, Japan), which included bioelectrical impedance analysis. The equipment used in this study has been found to be reliable (intraclass correlation coefficient ≥0.95–0.99) in men and women [39,40], and has been previously used in law enforcement populations [41]. Manufacturer guidelines were followed to record FM% [39], and the procedures used were also reported by Lockie et al. [41]. The age, height in cm, and sex of the LEO were entered into the device, and the LEO wore no shoes or socks. The LEO then stepped onto the scale with their feet positioned on the foot and heel electrodes, and were instructed to hold the display unit with both hands until their body mass was displayed on the screen. The display unit featured electrodes on the handles, and the hands were to be positioned on top of these electrodes. Once the LEO’s feet and hands were positioned on the appropriate electrodes (eight in total) [42], they stood upright and extended their arms so they were parallel to the ground. The scan was completed when the LEO’s body mass was displayed again. Proprietary equations from the device provided measurements of FM% [42]. The tester scrolled through the data on the display unit and recorded the appropriate variables. Body mass index (BMI) was derived via the calculation: body mass in kilograms (kg) ÷ (height in m)^2^.

### 2.5. Waist-to-Hip Ratio (WHR)

Waist and hip circumference are indicators of body fat distribution [43], and WHR has been used to assess law enforcement recruits [44]. The procedures described by Lockie et al. [44] were also adopted by the agency staff. A thin-line metric tape measure (Lufkin, Apex Tool Group, Sparks, MD, USA) was used to measure waist and hip circumference for all LEOs. Waist circumference was measured in cm at the narrowest part of the waist just superior to the naval. Hip circumference was measured at the greatest posterior extension of the hip. WHR was calculated by dividing waist circumference by hip circumference.

### 2.6. Grip Strength

Grip strength provided a measure of upper-body strength [45] and was measured by a hand grip dynamometer (Takei Scientific Instruments, Niigata, Japan). Further, grip strength has been used in a number of different law enforcement studies [14,20,41,44,46,47]. LEOs kept their testing arm by their side when standing throughout the assessment and squeezed the handle as hard as possible for approximately 2 s [14,20,44]. Two attempts were completed for each hand and recorded to the nearest kg, with the left hand tested first [44]. The best score for each hand was summed together to provide the combined grip strength score.

### 2.7. Sit-and-Reach

The sit-and-reach provided a measure of hamstring flexibility [48], and used procedures that have been detailed in the literature [21,49,50]. LEOs removed their shoes and sat with both feet flat against the sit-and-reach box and positioned their hands on top of each other (tips of the middle fingers aligned), with the palms down. The LEO then reached forward slowly and touched as far along the scale as possible, ensuring that the knees remained extended and held this position for 5 s. Three trials were performed, with the furthest reach distance used for analysis.

### 2.8. Push-Ups

Upper-body muscular endurance was assessed via a 60 s push-up test where LEOs completed as many repetitions as possible in this time period, and set procedures were followed [44,51,52,53]. A tester placed a fist on the floor directly under the chest of the LEO to ensure they descended to the correct depth. All female LEOs were partnered with a female tester. On the start command, the tester began the stopwatch and the LEO flexed their elbows and lowered themselves until their chests contacted the tester’s fist before they extended their elbows to return to the start position. LEOs performed as many push-ups as possible in 60 s, with the recorded result being the number of correctly completed repetitions.

### 2.9. Sit-Ups

Abdominal muscular endurance was assessed via a 60 s sit-up test where LEOs completed as many repetitions as possible in this time period [44,51,52,53]. The LEO laid on their back with their knees flexed to 90°, heels flat on the ground, and arms crossed over the chest. The feet were held in place by a tester who also counted the repetitions. On the start command, the LEO raised their shoulders from the ground while keeping their arms crossed over the chest and touched their elbows to their knees. The LEO then descended back down until their shoulder blades contacted the ground. LEOs completed as many repetitions as possible in 60 s, with the recorded result being the number of correctly completed repetitions.

### 2.10. YMCA Step Test

The YMCA step test was administered as a fitness assessment to measure aerobic capacity, and was administered via set procedures [54,55,56]. This test has been used to assess aerobic fitness in custody assistant recruits [56], highlighting its applicability for LEOs. The test was performed with approximately 12 inch (~31 cm) high bleacher seats used for the step on an indoor basketball court. Although it could have been beneficial to customize step heights to all LEOs, this was not feasible within the confines of patrol school. LEOs completed the step test in groups of 6–8, such that they could be paired up with a tester to measure their recovery heart rate (HR).

To complete the YMCA step test, LEOs stepped in time to a 96 beats per minute metronome continuously for 3 min. The beat was played from an iPad handheld device (Apple Inc., Cupertino, CA, USA) connected to a portable speaker (ION Block Rocker, Cumberland, RI, USA) positioned on a bleacher seat in front of the officers. Following the 3-minute time period, LEOs immediately sat on the step while recovery HR was manually taken by a staff member via the carotid or radial artery for 60 s [55,56,57].

### 2.11. Statistical Analysis

As secondary data were utilized in this study, G*Power software (v3.1.9.2, Universität Kiel, Kiel, Germany) was used to confirm post hoc that the sample size of 60 was sufficient for an analysis of covariance (ANCOVA) such that data could be interpreted with a small effect level of 0.4 [58], and a power level of 0.8 when significance was set at 0.05 [59]. The ANCOVA statistical analyses were computed using the Statistics Package for Social Sciences (Version 27.0; IBM Corporation, New York, NY, USA). Descriptive statistics (mean ± standard deviation [SD]) were calculated for each variable. The sample was divided into three groups: LEO ≤ 24 (LEOs who worked in custody for ≤24 months; *n* = 15); LEO 2547 (LEOs who worked in custody for 25–47 months; *n* = 24); and LEO 48+ (LEOs who worked in custody for ≥48 months; *n* = 21). These time periods have been used in previous research [12], and allowed for a relatively equitable distribution of LEOs across the groups. Levene’s test for equality of variances assessed the homogeneity of variance of the data, with significance set at *p* < 0.05. If data were found to be heterogeneous, the alpha level required for between-group significant interactions was adjusted to *p* < 0.01 to reduce Type I errors [12]. Observed power for the between-group comparisons was noted. A univariate ANCOVA was used to determine whether there were significant differences between the groups. Within these groups, the sexes were combined [21,44,56]. Nevertheless, sex was used as a covariate as previous research has illustrated between-sex differences in the physical performance of law enforcement personnel [14,20,22,52,60,61,62,63,64]. All variables except for age and height were also analyzed with age as an additional covariate [12], as age can influence body mass and fitness test performance [19,20,21,22,23]. If a significant interaction between the groups was found, a Bonferroni post hoc adjustment for multiple pairwise comparisons was adopted (*p* < 0.05). To provide an exploratory analysis and visualize the health and fitness of individual LEOs, scatter plots were also produced for the variables relative to the number of years spent working in custody using Microsoft Excel (Microsoft Corporation™, Redmond, WA, USA).

The second part of the analysis involved comparing the LEOs to normative data (relative to sex and age), and select data were profiled using Microsoft Excel (Microsoft Corporation™, Redmond, WA, USA). BP classifications were drawn from standards presented by Pescatello et al. [33]. BMI, sit-and-reach, and combined grip strength data were compared to normative data shown by Riebe et al. [34]. FM%, WHR, RHR, and recovery HR from the YMCA step test were compared to normative data presented by Ryan and Cramer [35]. Push-ups and sit-ups were not included in this part of the analysis as there was no established normative data that used the same methods as the staff from this agency.

## 3. Results

The order of the data presented follow this general grouping: descriptive data (age, height, and body mass); body composition (BMI, FM%, and WHR); clinical measures (RHR and BP); and physical fitness measures (grip strength, sit-and-reach, push-ups, sit-ups, and YMCA step test recovery HR). Homogeneity of variance data from Levene’s test for equality of variances, and the resulting alpha level used for the ANCOVA, are shown in Table 1. The observed power for the ANCOVA for each variable is also shown in Table 1.

Descriptive data for the health and fitness assessments are shown in Table 2. There was a significant interaction for age (F_2_ = 8.620, *p* = 0.001) and sit-ups (F_2_ = 5.443, *p* = 0.007), and both of these had high observed power (>0.8). The LEO ≤ 24 (*p* = 0.001) and LEO 2547 (*p* = 0.023) groups were significantly younger than the LEO 48+ group. The LEO 48+ group completed fewer sit-ups than the LEO 2547 (*p* = 0.006) and LEO ≤ 24 groups, although the difference with the LEO ≤ 24 group did not reach significance (*p* = 0.075). There were no significant between-group interactions for height (F_2_ = 0.447, *p* = 0.642), body mass (F_2_ = 0.752, *p* = 0.476), BMI (F_2_ = 0.499, *p* = 0.610), FM% (F_2_ = 0.223, *p* = 0.801), WHR (F_2_ = 3.056, *p* = 0.055), RHR (F_2_ = 0.395, *p* = 0.676), systolic BP (F_2_ = 1.195, *p* = 0.310), diastolic BP (F_2_ = 0.723, *p* = 0.490), combined grip strength (F_2_ = 0.788, *p* = 0.460), sit-and-reach (F_2_ = 0.850, *p* = 0.433), push-ups (F_2_ = 1.364, *p* = 0.264), and the YMCA step test recovery HR (F_2_ = 0.385, *p* = 0.682). Health and fitness data for individual LEOs relative to the number of years spent working in custody can be viewed in the scatter plots shown in the Supplementary Materials (Figure S1). There was a tendency for officers to be spread across the health and fitness variables relative to number of years working in custody, which reinforced the results calculated from the univariate ANCOVA.

**Figure 1:**
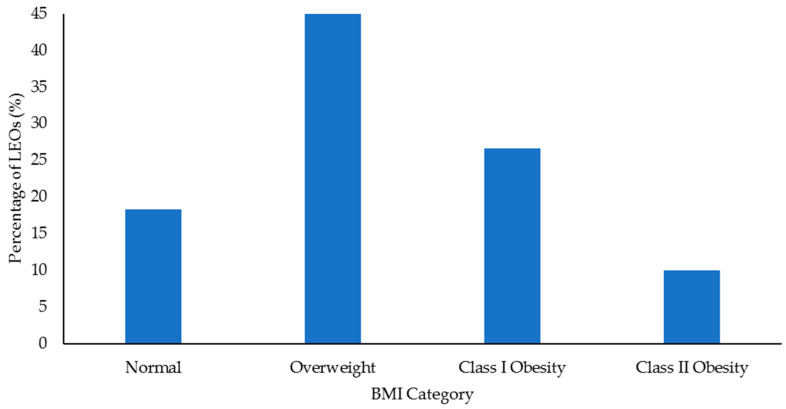
Percentage of law enforcement officers (LEOs) in this sample (*n* = 60) classified according to the body mass index (BMI) categories.

**Figure 2:**
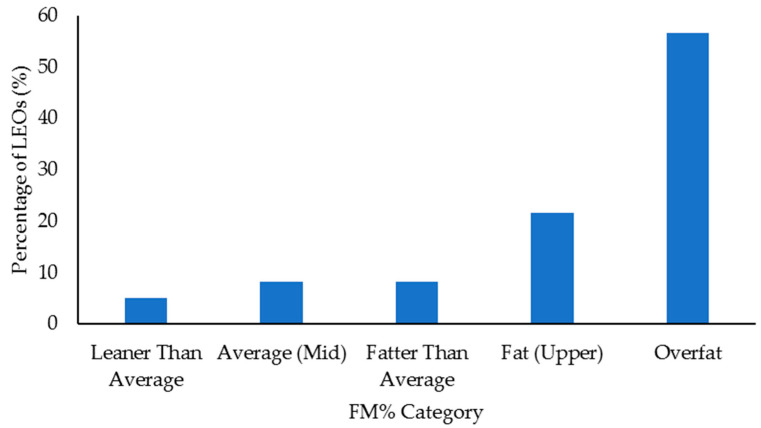
Percentage of law enforcement officers (LEOs) in this sample (*n* = 60) classified according to the percentage of fat mass (FM%) categories.

**Figure 3:**
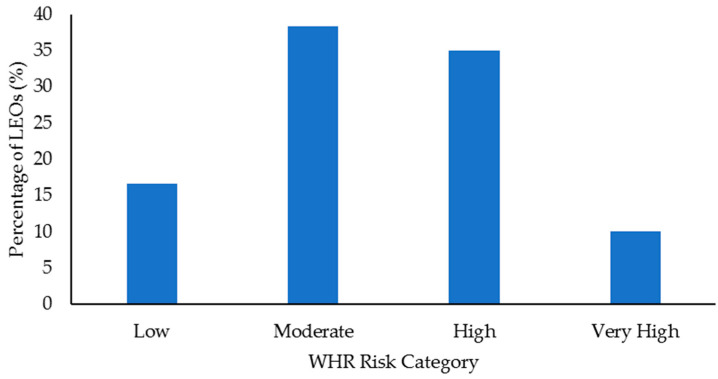
Percentage of law enforcement officers (LEOs) in this sample (*n* = 60) classified according to the waist-to-hip ratio (WHR) cardiovascular disease risk categories.

**Figure 4:**
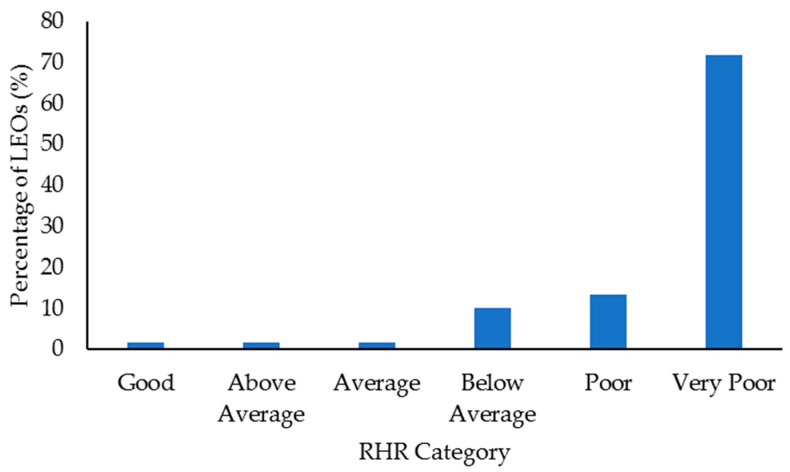
Percentage of law enforcement officers (LEOs) in this sample (*n* = 60) classified according to the resting heart rate (RHR) categories.

**Figure 5:**
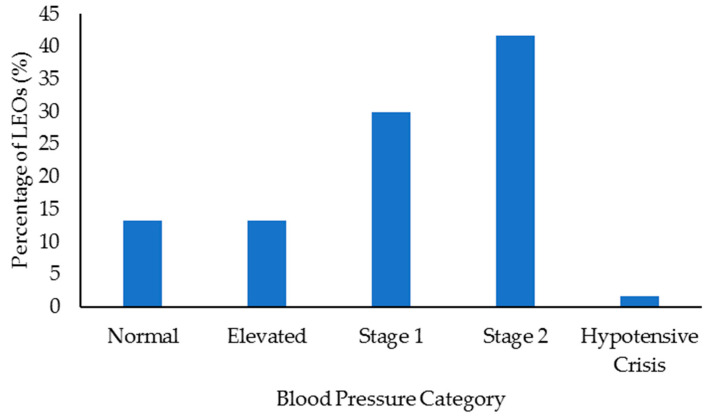
Percentage of law enforcement officers (LEOs) in this sample (*n* = 60) classified according to the blood pressure standards.

**Figure 6:**
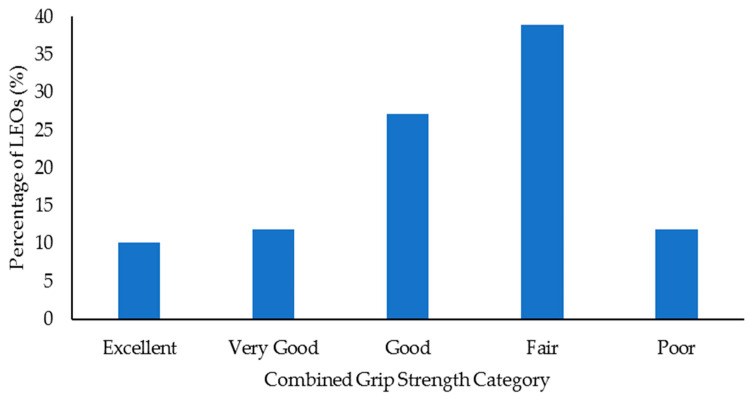
Percentage of law enforcement officers (LEOs) in this sample (*n* = 60) classified according to the combined grip strength categories.

**Figure 7:**
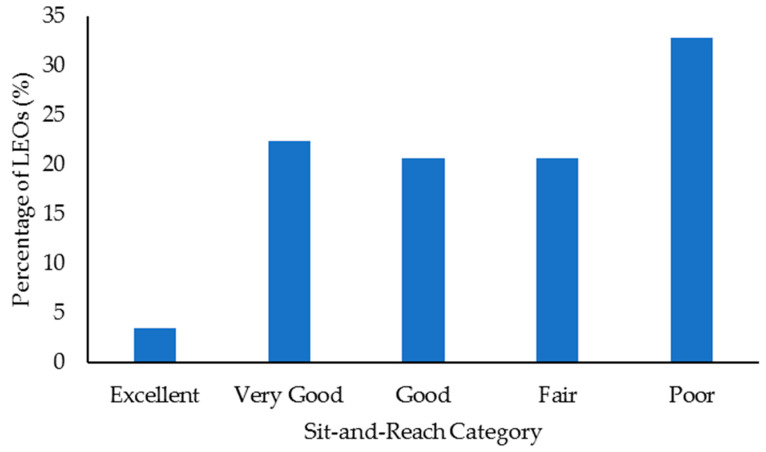
Percentage of law enforcement officers (LEOs) in this sample (*n* = 60) classified according to the sit-and-reach forward flexion categories.

**Figure 8:**
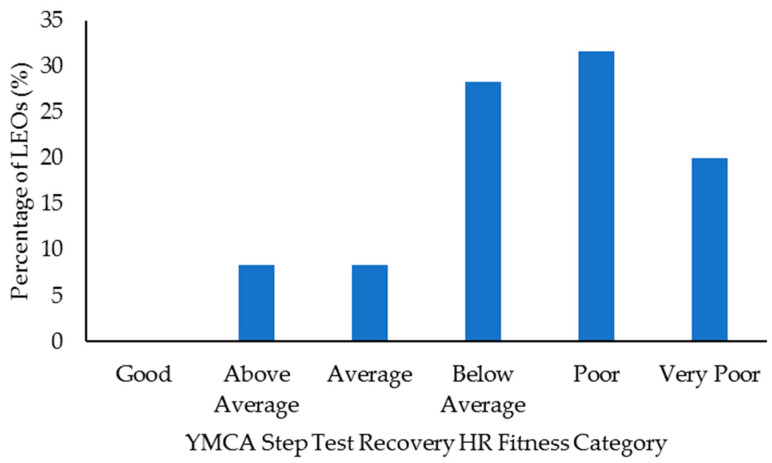
Percentage of law enforcement officers (LEOs) in this sample (*n* = 60) classified according to the YMCA step test recovery heart rate (HR) categories.

Select health and fitness assessment performance of officers following their custody assignment relative to normative data is shown in Figure 1, Figure 2, Figure 3, Figure 4, Figure 5, Figure 6, Figure 7 and Figure 8. Regarding BMI (Figure 1), 82% of the sample (*n* = 49) were categorized from overweight to class II obesity. Relative to their FM% (Figure 2), 87% of the sample (*n* = 52) were categorized as being fatter than average or above. Approximately 45% of the sample (*n* = 27) had a WHR that placed them at high or very high risk of cardiovascular disease (Figure 3). Over 70% of the sample (*n* = 43) had an RHR categorized as being very poor (Figure 4). Relative to BP (Figure 5), approximately 87% of the sample (*n* = 52) had a BP elevated above normal, with one classified as hypotensive crisis (this LEO was referred to medical staff at the agency but experienced no health issues). Almost 50% of the LEOs (*n* = 29) were categorized as having good-to-excellent grip strength; however, the largest number of LEOs (~39%; *n* = 23) was categorized as fair (Figure 6). Approximately 45% of the LEOs (*n* = 27) were good-to-excellent in the sit-and-reach, although 33% of the sample (*n* = 19) were categorized as having poor flexibility (Figure 7). Lastly, 80% of the sample (*n* = 48) were categorized as having below average to very poor aerobic fitness as measured by recovery HR following the YMCA step test (Figure 8).

## 4. Discussion

This study analyzed the influence of time spent working in custody on the health and fitness characteristics of LEOs. It was hypothesized that a longer time spent working in custody would result in poorer health and fitness. However, these results indicate that there was only one significant difference between groups, with the LEO 48+ group completing fewer sit-ups than the LEO 2547 group. Several trends towards poorer fitness in the LEO 48+ group were observed, which may reach significance with a larger sample size. What was more notable from the data analysis was that regardless of time spent working in custody, most of this sample of LEOs had relatively poor health and fitness characteristics. Indeed, many officers had higher risks of cardiovascular disease, which unfortunately is indicative of law enforcement populations [24]. Further to this, LEOs with poorer health and fitness characteristics relative to the general population could experience challenges in safely and effectively performing essential job tasks. The findings from this study have important implications for law enforcement staff, and the need for the provision of physical activity opportunities and education for personnel.

The LEO 48+ group were significantly older than both the LEO ≤ 24 and LEO 2547 groups. These results were expected, had a high observed power of 0.960, and have been shown previously by Lockie et al. [12] in their sample of LEOs. The current results indicated few significant differences in health and fitness between the groups. The LEO 48+ group did perform fewer repetitions in the 60 s sit-up test compared to the other groups (observed power of 0.827), specifically the LEO 2547 group. Orr et al. [19] previously detailed that male patrol officers completed ~7 fewer sit-ups in 60 s compared to age-matched cadets. The data from Orr et al. [19] provided some evidence of declines in abdominal muscular endurance in law enforcement personnel. As abdominal strength and endurance has been related to policing job-task performance [4,6,8], these data are potentially impactful. To provide a specific example, Lockie et al. [4] found that in law enforcement recruits, greater sit-up repetitions significantly (*p* ≤ 0.006) correlated with faster times in a 99-yard obstacle course (*r* = −0.208), 6-foot chain link fence climb (*r* = −0.175), and 500-yard run (*r* = −0.344). If an LEO experiences reduced abdominal strength and endurance following their time in custody, this could negatively impact their ability to perform the tasks required of them in a patrol position.

However, there were no other significant between-group differences in the health and fitness assessments. This was reinforced by the scatter plot data shown for each variable in the Supplementary Materials (Figure S1), whereby the healthiest and fittest LEOs did not always spend the least amount of time working in custody. This was counter to the study hypotheses, and to previous research indicating job-specific fitness declines in LEOs following time spent working in custody [12,13]. What these data would suggest is that irrespective of time spent working in custody, health and fitness characteristics could be similar amongst a sample of LEOs. Accordingly, it is important to categorize the health and fitness of LEOs relative to established normative data [33,34,35]. These data could highlight not only challenges for the individual officer relative to their overall health and well-being, but also potential job limitations if an LEO is deficient in a certain fitness quality. One of the chief concerns from the study results was that many of the LEOs in this sample exhibited poor health and fitness and may be at increased risk of cardiovascular disease.

Obesity is a risk factor for cardiovascular disease [30,31], and BMI, FM%, and WHR provided some measure of this in the LEOs. More than 80% of the LEOs (*n* = 49) had a BMI that classified them as overweight or obese. Approximately 87% (*n* = 52) of the 60 LEOs were classified as being fatter than average or greater, with ~57% of the LEOs (*n* = 34) being categorized as overfat. Just under 50% of this sample of LEOs (*n* = 27) had a WHR that placed them at high-to-very high risk of cardiovascular disease. Even with the limitations associated with BMI (e.g., it does not provide a direct measure of body composition) [65], when combined with the FM% and WHR data, these results are less than ideal. Indeed, these body fat profiles should be concerning to law enforcement personnel and command staff and reinforce the need for LEOs to complete some form of physical activity to improve their body composition. This could also include nutritional advice and education, as in conjunction with low physical activity, poor food choices are a major factor in obesity [66]. Further, the sedentary nature of working in custody could be compounded by the shift work and irregular work hours completed by law enforcement personnel, as this can also lead to poorer dietary choices [67,68].

Both elevated RHR [26,27,28] and BP [29] are indicators of increased cardiovascular disease risk. Approximately 72% of the 60 LEOs (*n* = 43) had a very poor RHR, and ~87% of the LEOs (*n* = 52) had elevated BP (or worse). It should be acknowledged that these measurements were not taken first thing in the morning, which is generally recommended [38], so may not be the best representation of the overall health of the LEOs. Further to this, law enforcement training situations can increase the stress (indicated by elevated HR responses) experienced by individuals [69,70]. Additionally, 80% of the LEO sample (*n* = 48) had an RHR that fell within the normal adult range of 60–100 bpm, and resting HR can vary greatly (from 40 to 109 bpm) between adults [71]. Nonetheless, when taken together with the other results in this study (as will be discussed, FM%, WHR, and the YMCA step test recovery HR), it could be surmised that most LEOs had an RHR and BP higher than what would be preferred. Reducing RHR and BP could be key in reducing the risk of an officer experiencing a cardiac event [26,29,71]. Following a meta-analyses of the literature, Cornelissen and Fagard [72] stated that aerobic endurance training could reduce BP in adults, in addition to other factors that contribute to cardiovascular disease (e.g., systemic vascular resistance, fat mass). Accordingly, aerobic exercise could be used to help reduce RHR and BP in LEOs and alleviate some of the negative impacts that could result from working in custody. These data suggest that law enforcement agencies and command staff should create opportunities for exercise where appropriate for LEOs working in custody. Access to trained professionals such as strength and conditioning coaches could also aid this process [73], as law enforcement personnel could have direct contact with individuals that can design specific programs that can fit within their life and job demands.

Approximately 50% the sample of LEOs (*n* = 29) were categorized as good-to-excellent with their grip strength, with most (~39% of the sample; *n* = 23) in the fair category. As inmate restraint is a necessary job task in custody, and grip strength is important for this task [16], most of the officers may have been able to maintain this strength quality. Grip strength also contributes to several essential patrol tasks, such as defensive tactics [46], firearm use [46,47], and dragging an incapacitated individual [7]. LEOs deficient in their grip strength should seek to perform appropriate resistance training to enhance this quality. In something of a contrast to grip strength, a much higher sample of the LEOs were categorized as poor in the sit-and-reach flexibility test (~33% of the sample; *n* = 19). Although the sit-and-reach test is limited to measuring hamstring flexibility [48], these results are still important. Flexibility is important for conducting searches in different locations (e.g., cells, vehicles, rooms in houses) [14,15,16] and for manual handling [74]. Greater hamstring flexibility has also been found to decrease overuse injuries in military trainees [75], so this could have some impact on LEOs as they transition from custody to patrol job duties. Specific flexibility training could be used to enhance this quality in LEOs [76], or it could be combined with other training modalities (e.g., resistance training) [77]. Utilization of strength and conditioning coaches could be beneficial for LEOs who may not have the training or knowledge to design appropriate programs to achieve improvements in strength and flexibility concurrently.

Aerobic capacity is an important contributor to policing tasks, including load carriage [78], and endurance- and running-based activities [4,5,6,8]. Further, better aerobic fitness can lower the risk of cardiovascular disease in men and women [79]. Unfortunately, 80% of the 60 LEOs in this study sample (*n* = 48) had a recovery HR following the YMCA step test that was categorized as below average-to-very poor. As the YMCA step test provided a measure of aerobic fitness [55], this is not ideal. The recovery HR results fall in line with those from Orr et al. [19], who detailed that male patrol officers had a 16% increase in time to complete a 2.4 km run (which measured aerobic fitness) compared to cadets. As several studies have indicated that with appropriate physical training, aerobic capacity can be improved over the course of a training academy [3,10,11], aerobic fitness declines likely occurred during the custody period for these LEOs. The prevalence of sedentary activities performed during custody work could have negatively impacted aerobic capacity in these officers. Providing opportunities to complete physical activity and aerobic conditioning during the custody period, and education of the importance of this relative to job performance and general health, could be beneficial for improving the health and fitness of LEOs. As noted, access to strength and conditioning coaches could benefit law enforcement personnel seeking to improve their aerobic fitness.

Categorical data specific to the methods completed for push-ups and sit-ups were not available within the literature. Orr et al. [19] did suggest that upper-body and abdominal muscular endurance tended to decline in patrol officers compared to cadets. However, the LEO ≤ 24 and LEO 2547 officers in this study had similar push-up and sit-up performance compared to recruits from the same agency at the start of academy who then proceeded to graduate (push-ups = 44.59 ± 15.40 repetitions; sit-ups = 35.96 ± 9.09 repetitions) [52]. Further, the LEO ≤ 24 and LEO 2547 groups had mean push-up data that were superior to those of the state patrol officers from Dawes et al. [20] (males = 39.09 ± 15.61 repetitions; females = 24.24 ± 11.63 repetitions), while the sit-up data were similar (males = 34.46 ± 10.29 repetitions; females = 31.06 ± 9.52 repetitions). The LEO 48+ group had push-up and sit-up data that were superior to the female state patrol officers from Dawes et al. [20], but not the male officers. As previously discussed, the LEO 48+ group did complete less sit-ups than the other groups, so it may be that abdominal muscular endurance became more of an issue the longer an officer spent working in custody. As both upper-body and abdominal endurance contribute to important patrol job tasks [4,5,8], LEOs should strive to maintain these qualities as best they can. Appropriate resistance training should be able to achieve this and could be best facilitated via access to a strength and conditioning coach as part of the law enforcement agency [73]. Additionally, as the procedures adopted by this agency are prevalent within law enforcement populations [44,51,52,53], future research should establish categorical data for the push-up and sit-up assessments in male and female LEOs.

There are certain study limitations to this study that should be noted. This study utilized only one patrol school class, which featured 60 LEOs (with groups of 15, 24, and 21 officers as part of the analysis). The sample size was relatively small, especially when considering the size of large law enforcement agencies (e.g., the largest sheriff’s department in the world employs 18,000 personnel) [80,81]. More research on a greater number of law enforcement personnel is required. Nonetheless, given the challenges when conducting research with sworn personnel (e.g., time constraints, physical demands of testing) [82,83], this study is an important addition to the literature. Additionally, this research was cross-sectional in nature. Similar to Lockie et al. [13], it would be of benefit to track the health and fitness of LEOs from their time in academy, to their time spent working in custody and then patrol. This could provide a more specific picture as to the effects of the occupation on the health and fitness of an LEO. Other body composition equipment (e.g., InBody BIA technology) may provide a more detailed analysis of lean body and fat mass [84,85], and it could be beneficial to incorporate these into future studies on the health and fitness of law enforcement personnel. Age could have impacted the health and fitness characteristics of the sample in this study [20,21,22,23]. Indeed, as reflected in the current data and previous research [12], the personnel who worked in custody for longer tended to be older. Nonetheless, and similar to Lockie et al. [12], this was one of the reasons why age was included as a covariate in this study. Grip strength was the only measure of maximal strength in this study. As maximal strength is important for law enforcement job tasks [7,82,86,87,88], future research should incorporate different maximal strength assessments to measure this quality in their incumbent personnel, including tests such as the bench press, back squat, barbell or hexagonal bar deadlift, and leg/back chain dynamometer (depending on what is most appropriate for the personnel) [7,20,78,82,86,88]. As the fitness of personnel can vary between different agencies [89], it is important for individual agencies to develop their own data banks that categorize the health and fitness of their workforce.

## 5. Conclusions

The results from this study indicated that there were limited differences in health and fitness between LEOs who had worked in custody facilities for shorter or longer time periods. Although the LEO 48+ group completed fewer sit-ups compared to the LEO 2547 group, this was the only significant difference. Irrespective of time spent working in custody, this sample of LEOs tended to display poor health and fitness when compared to normative, categorical data. For example, ~72% of the LEOs (43 out of 60) were categorized as having a very poor RHR, while ~87% (52 LEOs) had elevated blood pressure. Approximately 82% of the sample (49 LEOs) were categorized as overweight-to-class II obesity according to their BMI, and ~87% (52 LEOs) were fatter than average or above relative to their FM%. Almost half the sample (27 LEOs) had a WHR that indicated increased cardiovascular disease risk, and 80% (48 LEOs) were categorized as having an average-to-very poor recovery HR following the YMCA step test. These results suggest that LEOs should remain physically active to at the very least maintain their health and fitness following academy, especially given the population’s cardiovascular disease risk. Command staff at law enforcement agencies should provide opportunities for physical activity for LEOs working in custody, in addition to education about exercise and diet. Access to strength and conditioning coaches would also be beneficial for LEOs working in custody, especially before they transition into a patrol position. This would aid in officers receiving specific and appropriate training programs that could improve their aerobic fitness, muscular strength and endurance, flexibility, and general health.

## Figures and Tables

**Table 1:** Homogeneity of variance data from Levene’s test for equality of variances, and the resulting *p* value used for the ANCOVA, for age, height, body mass, body mass index, fat mass percentage, waist-to-hip ratio, resting heart rate, systolic and diastolic blood pressure, combined grip strength, sit-and-reach, push-ups, sit-ups, and recovery heart rate from the YMCA step test in law enforcement officers.

Variables	F_2_ Value	*p* Value	Assumption	ANCOVA Alpha Level	Observed Power
Age	0.682	0.509	Homogenous	*p* < 0.05	0.960
Height	0.926	0.402	Homogenous	*p* < 0.05	0.119
Body Mass	0.131	0.878	Homogenous	*p* < 0.05	0.171
Body Mass Index	0.025	0.975	Homogenous	*p* < 0.05	0.128
Fat Mass Percentage	0.230	0.796	Homogenous	*p* < 0.05	0.083
Waist-to-Hip Ratio	2.819	0.068	Homogenous	*p* < 0.05	0.568
Resting Heart Rate	0.546	0.582	Homogenous	*p* < 0.05	0.111
Systolic Blood Pressure	1.321	0.275	Homogenous	*p* < 0.05	0.251
Diastolic Blood Pressure	0.573	0.567	Homogenous	*p* < 0.05	0.166
Combined Grip Strength	1.782	0.178	Homogenous	*p* < 0.05	0.178
Sit-and-Reach	3.188	0.049	Heterogeneous	*p* < 0.01	0.188
Push-ups	0.805	0.452	Homogenous	*p* < 0.05	0.282
Sit-ups	2.060	0.137	Homogenous	*p* < 0.05	0.827
YMCA Recovery Heart Rate	0.723	0.490	Homogenous	*p* < 0.05	0.109

**Table 2:** Descriptive data (mean ± SD) for age, height, body mass, body mass index (BMI), fat mass percentage, waist-to-hip ratio, resting heart rate, systolic and diastolic blood pressure (BP), combined grip strength, sit-and-reach, push-ups, sit-ups, and recovery heart rate (HR) from the YMCA step test in law enforcement officers who had spent ≤24 months (LEO ≤ 24), 25–47 months (LEO 2547), and ≥48 months (LEO 48+) working in custody.

Variables	LEO ≤ 24 (*n* = 15)	LEO 2547 (*n* = 24)	LEO 48+ (*n* = 21)
Age (years)	28.07 ± 5.52	30.67 ± 5.20	35.71 ± 5.68 *^,§^
Height (m)	1.72 ± 0.07	1.74 ± 0.08	1.72 ± 0.09
Body Mass (kg)	84.63 ± 12.72	86.27 ± 15.28	87.78 ± 19.42
BMI (kg·m^−2^)	28.60 ± 3.81	28.29 ± 3.44	29.38 ± 4.29
Fat Mass Percentage (%)	27.95 ± 7.87	27.56 ± 6.67	30.42 ± 6.02
Waist-to-Hip Ratio	0.87 ± 0.07	0.86 ± 0.07	0.88 ± 0.08
Resting Heart Rate (bpm)	90.20 ± 10.64	89.43 ± 11.78	88.25 ± 15.61
Systolic BP (mmHg)	126.47 ± 14.49	132.10 ± 13.15	134.62 ± 18.97
Diastolic BP (mmHg)	82.53 ± 9.18	87.90 ± 10.78	87.75 ± 10.15
Combined Grip Strength (kg)	88.34 ± 9.42	91.71 ± 17.77	81.15 ± 24.07
Sit-and-Reach (cm)	26.25 ± 8.92	28.76 ± 7.64	27.13 ± 6.91
Push-ups (repetitions)	43.47 ± 13.35	42.10 ± 14.76	33.08 ± 14.90
Sit-ups (repetitions)	34.07 ± 5.41	35.71 ± 9.13	27.25 ± 8.60 ^§^
YMCA Recovery HR (bpm)	123.13 ± 10.26	119.95 ± 11.81	120.08 ± 12.55

* Significantly (*p* < 0.05) different from LEO ≤ 24. ^§^ Significantly (*p* < 0.05) different from LEO 2547.

## Supplementary Materials

The following are available online at https://www.mdpi.com/article/10.3390/ijerph18179297/s1, Figure S1: Scatter plot data for body mass index, fat mass percentage, waist-to-hip ratio, resting heart rate, systolic and diastolic blood pressure, combined grip strength, sit-and-reach, push-ups, sit-ups, and recovery heart rate from the YMCA step test in law enforcement officers relative to years spent working in custody.
