# Supplementary material for: How Does Time Spent Working in Custody Influence Health and Fitness Characteristics of Law Enforcement Officers?
Source: Int J Environ Res Public Health. 2021 Sep 3;18(17):9297. doi: 10.3390/ijerph18179297 (PMC8431568; doi:10.3390/ijerph18179297)
Supplement: Supplementary file 1 [file ijerph-18-09297-s001.zip › ijerph-1244971-supplementary.pdf]

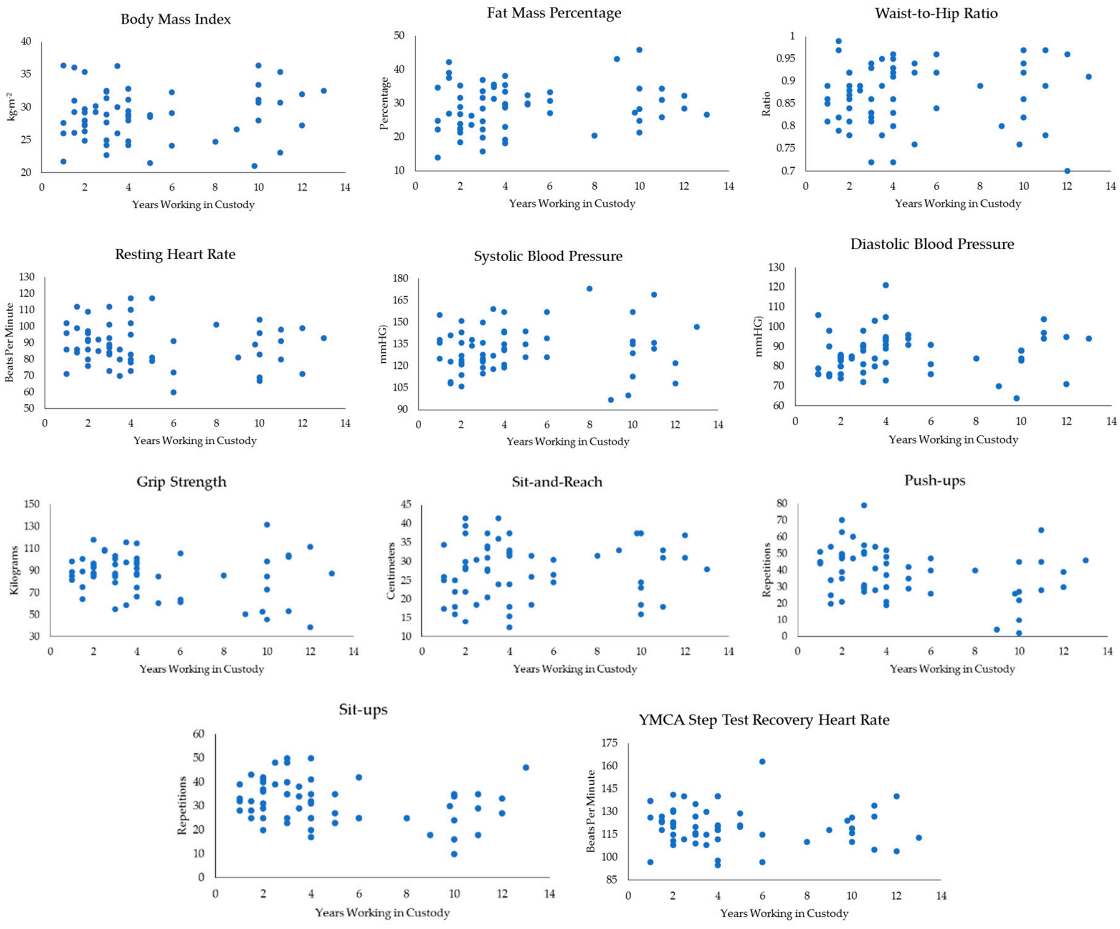

Figure S1. Scatter plot data for body mass index, fat mass percentage, waist-to-hip ratio, resting heart rate, systolic and diastolic blood pressure, combined grip strength, sit-and-reach, push-ups, sit-ups, and recovery heart rate from the YMCA step test in law enforcement officers relative to years spent working in custody.
